# A Pilot Study of Pembrolizumab in Combination With Y90 Radioembolization in Subjects With Poor Prognosis Hepatocellular Carcinoma

**DOI:** 10.1093/oncolo/oyad331

**Published:** 2024-02-07

**Authors:** Shawn Yu, Menggang Yu, Barry Keane, David M Mauro, Paul R Helft, William P Harris, Hanna K Sanoff, Matthew S Johnson, Bert O’Neil, Autumn Jackson McRee, Ashwin Somasundaram

**Affiliations:** Department of Medicine, The University of North Carolina at Chapel Hill, Chapel Hill, NC, USA; Department of Biostatistics, University of Michigan, Ann Arbor, MI, USA; Department of Medicine, The University of North Carolina at Chapel Hill, Chapel Hill, NC, USA; Department of Medicine, The University of North Carolina at Chapel Hill, Chapel Hill, NC, USA; Department of Medicine, Indiana University Simon Cancer Center, Indianapolis, IN, USA; Department of Medicine, University of Washington/FHCC, Seattle, WA, USA; Department of Medicine, The University of North Carolina at Chapel Hill, Chapel Hill, NC, USA; Department of Radiology and Imaging Sciences, Indiana University, Indianapolis, IN, USA; Community Health Network, Indianapolis, IN, USA; Janssen Research & Development, LLC, Spring House, PA, USA; Department of Medicine, The University of North Carolina at Chapel Hill, Chapel Hill, NC, USA

**Keywords:** hepatocellular carcinoma, pembrolizumab, immunotherapy, glass Y90 radioembolization, TARE

## Abstract

**Background:**

Combination checkpoint inhibition therapy with yttrium-90 (Y90) radioembolization represents an emerging area of interest in the treatment of advanced hepatocellular carcinoma (HCC). HCRN GI15-225 is an open-label, single-arm multicenter, pilot study (NCT03099564).

**Methods:**

Eligible patients had poor prognosis, localized HCC defined as having portal vein thrombus, multifocal disease, and/or diffuse disease that were not eligible for liver transplant or surgical resection. Patients received pembrolizumab 200 mg intravenously every 3 weeks in conjunction with glass yttrium-90 (Y90) radioembolization TheraSphere. Primary endpoint was 6-month progression-free survival (PFS6) per RECIST 1.1. Secondary endpoints included time to progression (TTP), objective response rate (ORR), overall survival (OS), and safety/tolerability.

**Results:**

Between October 23, 2017 and November 24, 2020, 29 patients were enrolled: 2 were excluded per protocol. Fifteen of the remaining 27 patients were free of progression at 6 months (55.6%; 95% CI, 35.3-74.5) with median PFS 9.95 months (95% CI, 4.14-15.24) and OS 27.30 months (95% CI, 10.15-39.52). One patient was not evaluable for response due to death; among the remaining 26 patients, ORR was 30.8% (95% CI, 14.3-51.8) and DCR was 84.6% (95% CI, 65.1-95.6).

**Conclusion:**

In patients with localized, poor prognosis HCC, pembrolizumab in addition to glass Y90 radioembolization demonstrated promising efficacy and safety consistent with prior observations (ClinicalTrials.gov Identifier: NCT03099564; IRB Approved: 16-3255 approved July 12, 2016).

## Teaser

This pilot study explored whether using immunotherapy drugs in combination with locoregional therapies for patients with non-metastatic hepatocellular carcinoma is safe and beneficial, using pembrolizumab every 3 weeks starting 1 week before initial Y90 radioembolization.

**Lessons Learned**

- Pembrolizumab therapy in combination with glass Y90 radioembolization was safe and tolerable in patients with advanced, poor prognosis hepatocellular carcinoma (HCC) with preserved liver function.
- Pembrolizumab therapy in combination with glass Y90 radioembolization provided durable antitumor activity with promising progression-free survival and overall survival in patients with advanced, poor prognosis HCC.

## Discussion

Liver cancer is the sixth most commonly diagnosed cancer and the third leading cause of cancer death worldwide; hepatocellular carcinoma (HCC) accounts for 75%-85% of such diagnoses.^1^ Early-stage HCC is amenable to locoregional treatments including surgical resection, radiofrequency ablation, transarterial chemoembolization (TACE), Y90 radioembolization, and liver transplantation.^2,3^ Traditionally, locoregional therapies such as Y90 radioembolization have been limited to intermediate stage HCC.^4^ In a randomized controlled trial involving 45 patients with early to intermediate stage HCC, Salem et al demonstrated that patients randomized to Y90 radioembolization had significantly improved median time to progression (TTP) but similar median overall survival (OS) compared with conventional TACE. However, benefits of Y90 radioembolization did not extend to patients with advanced stage HCC or those with localized high-risk “poor prognosis” HCC.^5-7^ High-risk features of localized poor prognosis HCC include portal vein thrombosis (PVT), multifocal disease, or macrovascular invasion (MVI). Patients with localized high-risk HCC treated with Y90 therapy had a median OS of 13.3 months for Child-Pugh A (CP-A) and 6.9 months for Child-Pugh B7 (CP-B7) patients.^6,7^

**Figure 1.**
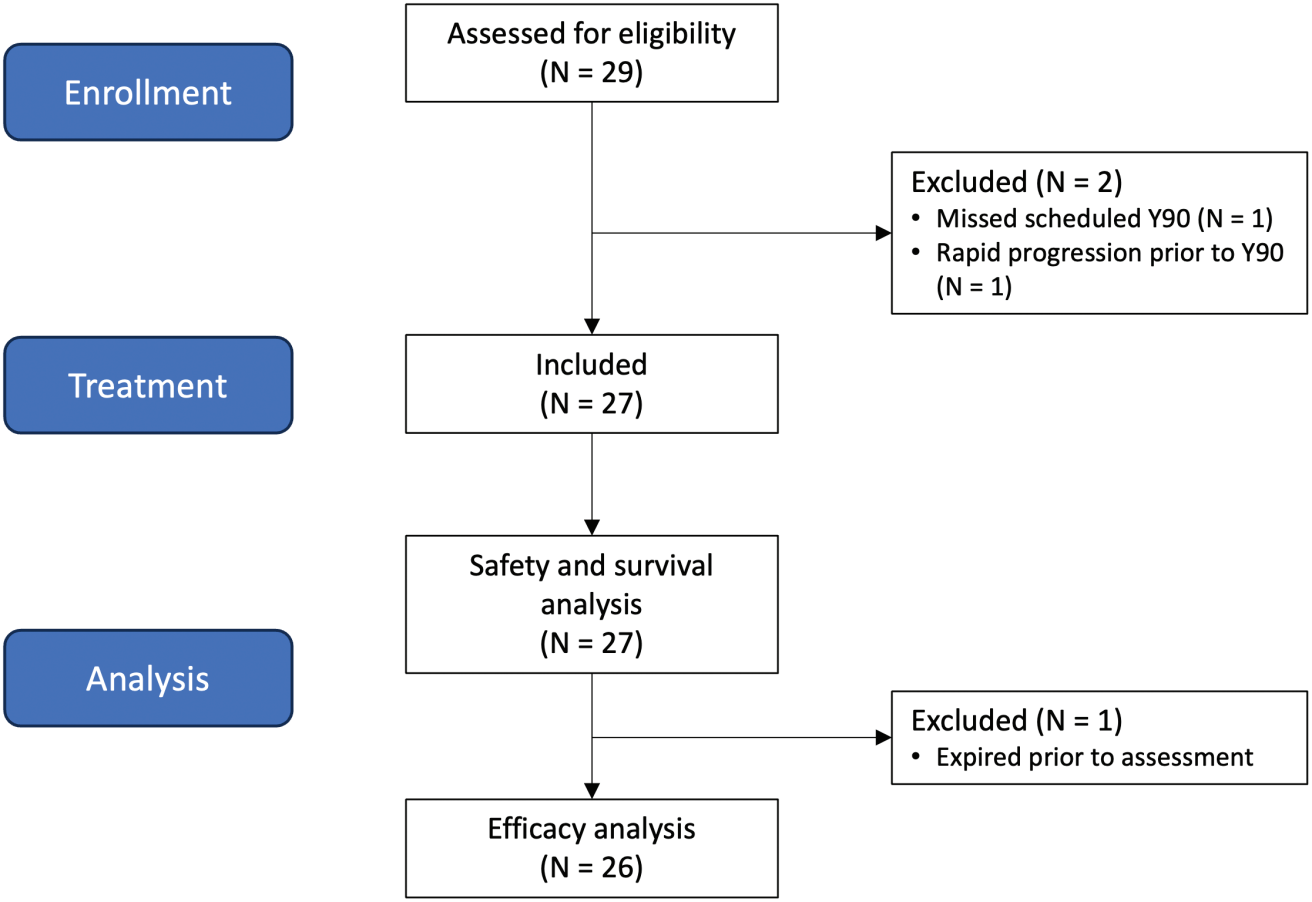
Patient flow diagram.

More recently, developments in immunotherapy have introduced nivolumab,^8,9^ nivolumab plus ipilimumab,^10^ pembrolizumab,^11-14^ atezolizumab plus bevacizumab,^15^ and sintilimab plus a bevacizumab biosimilar^16^ in the management of advanced HCC. This pilot study explores whether using immunotherapy drugs in combination with locoregional therapies for patients with non-metastatic HCC is safe and beneficial, using pembrolizumab every 3 weeks starting 1 week before initial Y90 radioembolization, as depicted in Fig. 1.

## Trial Information

**Table:** 

Disease	Hepatocellular carcinoma—HCC
Stage of disease/treatment	Locally advanced
Prior therapy	No designated number of regimens; no prior Y90 radioembolization permitted
Type of study	Pilot study, single arm
Primary endpoint	Progression-free survival at 6 months
Secondary endpoints	Time to progression, objective response rate, overall survival, safety/tolerability
Investigator’s analysis	Active and should be pursued further

## Additional Details of Endpoints or Study Design

HCRN GI15-225 is a multicenter, open-label, single-arm, pilot study conducted in 3 institutions: Fred Hutchinson Cancer Research Center (Seattle, WA), Indiana University Melvin and Bren Simon Comprehensive Cancer Center (Indianapolis, IN), and University of North Carolina Lineberger Comprehensive Cancer Center (Chapel Hill, NC).

### Patients and Eligibility

Eligible patients had locally advanced HCC, as defined by tissue diagnosis or elevated alpha-fetoprotein (AFP) > 400 ng/mL with compatible mass on imaging demonstrating both arterial hypervascularity and delayed washout. Patients with evidence of extrahepatic metastatic disease were excluded. Eligible patients had poor prognosis HCC, as defined as having portal vein involvement, multifocal disease, and/or diffuse disease. Finally, eligible patients were Eastern Cooperative Oncology Group (ECOG) performance status 0-1; Child-Pugh Cirrhotic Status A or B with maximum score of 7; disease amenable to embolization in 1 or 2 procedures; ineligibility for, or refusal of surgical resection or liver transplants; and no prior Y90 radioembolization. Full inclusion and exclusion criteria are provided in Supplementary Appendix.

### Treatment

After enrollment, patients underwent mandatory pretreatment biopsy, followed by pembrolizumab 200 mg intravenous every 3 weeks. Glass Y90 radioembolization occurred 7-10 days after the first dose of pembrolizumab. In patients requiring a second dose of Y90 radioembolization for bilobar disease, a second dose was administered within 4 weeks of the initial procedure, after cycle 2 and at least 1 week prior to cycle 3 of pembrolizumab. Dosimetry of Y90 was performed for each individual patient at the discretion of the authorized user. Treatment ranged from a single segment to whole liver. Based on patient anatomy and disease burden, the number of sessions and number of separate radioembolization sites per session were determined. Dose was optimized to not exceed approved standards nor exceed acceptable doses. For single lobe treatments, a dose of greater than 130 Gy was often targeted. Patients received pembrolizumab every 3 weeks and one or 2 doses of Y90 radioembolization as described above for up to 24 months or until disease progression, unacceptable toxicity, study withdrawal, or death. Imaging was conducted every 9 weeks to assess for tumor response and disease progression.

### Endpoints and Assessments

Primary criteria used for duration of response was Response Evaluation Criteria in Solid Tumors (RECIST) version 1.1. As secondary criteria, disease was also evaluated using modified RECIST (mRECIST) for HCC. Adverse events were assessed using NCI Common Terminology Criteria for Adverse Events (NCI CTCAE) v5 during treatment, at 30 days after completion of treatment, and at 3-month intervals thereafter for up to 2 years. The primary endpoint was the proportion of patients alive and progression-free 6 months after treatment initiation (6-month progression-free survival, or PFS6), assessed according to RECIST 1.1 by imaging review. This study has not been powered for formal statistical testing. However, the precision of our study’s 6-month PFS rate has been estimated in terms of the length of the half-width of the one-sided exact 95% confidence interval. We note that the distance from each proportion (*p*) to each lower bound is ~0.17 (with rounding). Published studies show that current treatment regimens lead to a median PFS of approximately 6 months (or *p* = 0.5 at 6 months). Clinically relevant improvements in median PFS would be an increase to 8, 9, or 10 months. In terms of 6-month PFS rates, this represents *p* = 0.59, 0.63, or 0.66, respectively (assuming an exponential distribution). We planned to accrue a total of 30 evaluable subjects at the rate of 2-3 per month for approximately 1 year. In particular, based on a one-sided one-arm log-rank test (Table 1), we can see the resulting powers at *N* = 30 and “Follow-up=24 months,” for testing PFS of 6 months vs. PFS of 8, 9, 10 months, respectively, are all exceeded by *N* = 29 and “Follow-up=36 months.” Secondary endpoints included time to progression (TTP; time from initiation of treatment to first documented disease progression); objective response rate (ORR; proportion of patients achieving confirmed complete response or partial response), assessed according to RECIST 1.1; overall survival (OS; time from initiation of treatment to death from any cause); and safety/tolerability.

**Table 1.** Numeric results for the one-sided, one-sample Logrank test.

Power	*N*	Follow-up, months	Null PFS, months	Alt PFS, months	Alpha
0.4352	30	24	6	8	0.050
0.6633	30	24	6	9	0.050
0.8038	30	24	6	10	0.050
0.4416	29	36	6	8	0.050
0.6678	29	36	6	9	0.050
0.8050	29	36	6	10	0.050

## Drug Information

**Table:** 

Generic/working name	Pembrolizumab
Company name	Merck & Co.
Drug type	Antibody
Drug class	Immune therapy
Dose	200 mg per flat dose
Unit	Milligrams
Route	Intravenous
Schedule of administration	Q3W

**Table:** 

Patient Characteristics	
Number of patients, male	24
Number of patients, female	3
Stage	Advanced stage (BCLC-C)
Age: median	66 years
Number of prior systemic therapies: median (range)	Not collected
Performance status: ECOG	0: 13 1: 14 2: 0 3: 0 4: 0

**Table:** 

Primary Assessment Method: Study Treatment	
Number of patients screened	29
Number of patients enrolled	29
Number of patients evaluable for toxicity	27
Number of patients evaluated for efficacy	26
Evaluation method	RECIST 1.1
Response assessment, CR	0 (0%)
Response assessment, PR	8 (30.8%)
Response assessment, SD	14 (53.8%)
Response assessment, PD	4 (15.4%)
Median duration assessments, PFS	9.95 months (CI: 4.14-15.24)
Median duration assessments, TTP	9.95 months (CI: 4.14-15.24)
Median duration assessments, OS	27.30 months (CI: 10.15-39.52)
Duration of treatment	5.5 months

## Outcome Notes

### Patients

Between October 23, 2017 and November 24, 2020, 29 patients were enrolled across 3 institutions. Twenty-seven patients received at least one dose of pembrolizumab and one dose of glass Y90 radioembolization; 2 patients (7%) did not and were excluded from analysis per protocol (one missed his scheduled Y90 radioembolization procedure and one was found to have rapid progression prior to receiving Y90 radioembolization). The median age was 66 years (range, 33-79). Most of the patients were male (88.9%, 24/27), Caucasian (85.2%, 23/27), Child-Pugh A (96%, 26/27), and treated with radioembolization only once (74%, 20/27). Underlying etiology of ESLD was not collected. At the time of data cutoff, 2 patients remained on treatment and 25 discontinued treatments. The most common reasons for discontinuation were progressive disease in 13 (48.1%) patients and adverse effects in 5 (18.5%) patients. Baseline characteristics are shown in Table 2.

**Table 2.** Baseline demographics.

Characteristics	Category/statistics	Count/value	Percent
Sex	Female	3	11
	Male	24	89
Race	Caucasian	23	85
	African American	1	4
	American Indian or Alaska Native	1	4
	Unknown	2	7
Ethnic	Hispanic or Latino	2	7
	Non-Hispanic	22	81
	Unknown	3	11
ECOG PS	0	13	48
	1	14	52
Age (years)	Median	66	
	Min	33	
	Max	79	
	Mean	65.2	
	SD	9.26	
Child-Pugh	A	26	96
	B7	1	4

### Efficacy

**Figure 2.**
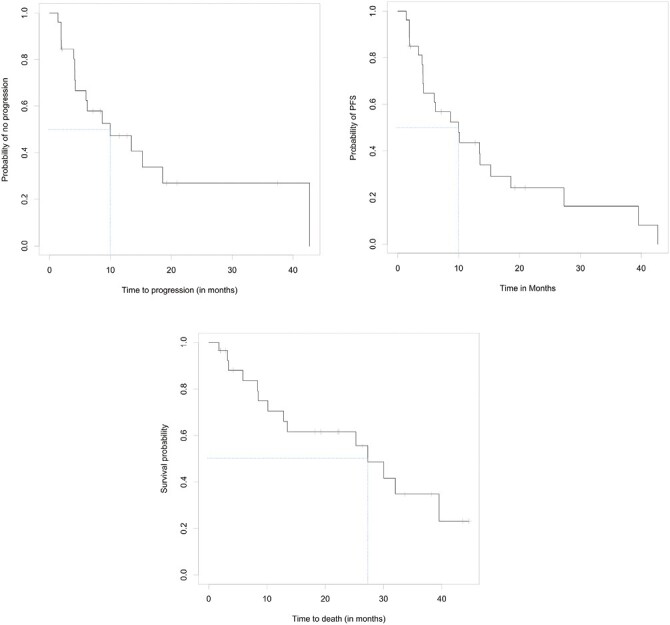
Kaplan–Meier curves for TTP (top), PFS (middle), and OS (bottom).

**Figure 3.**
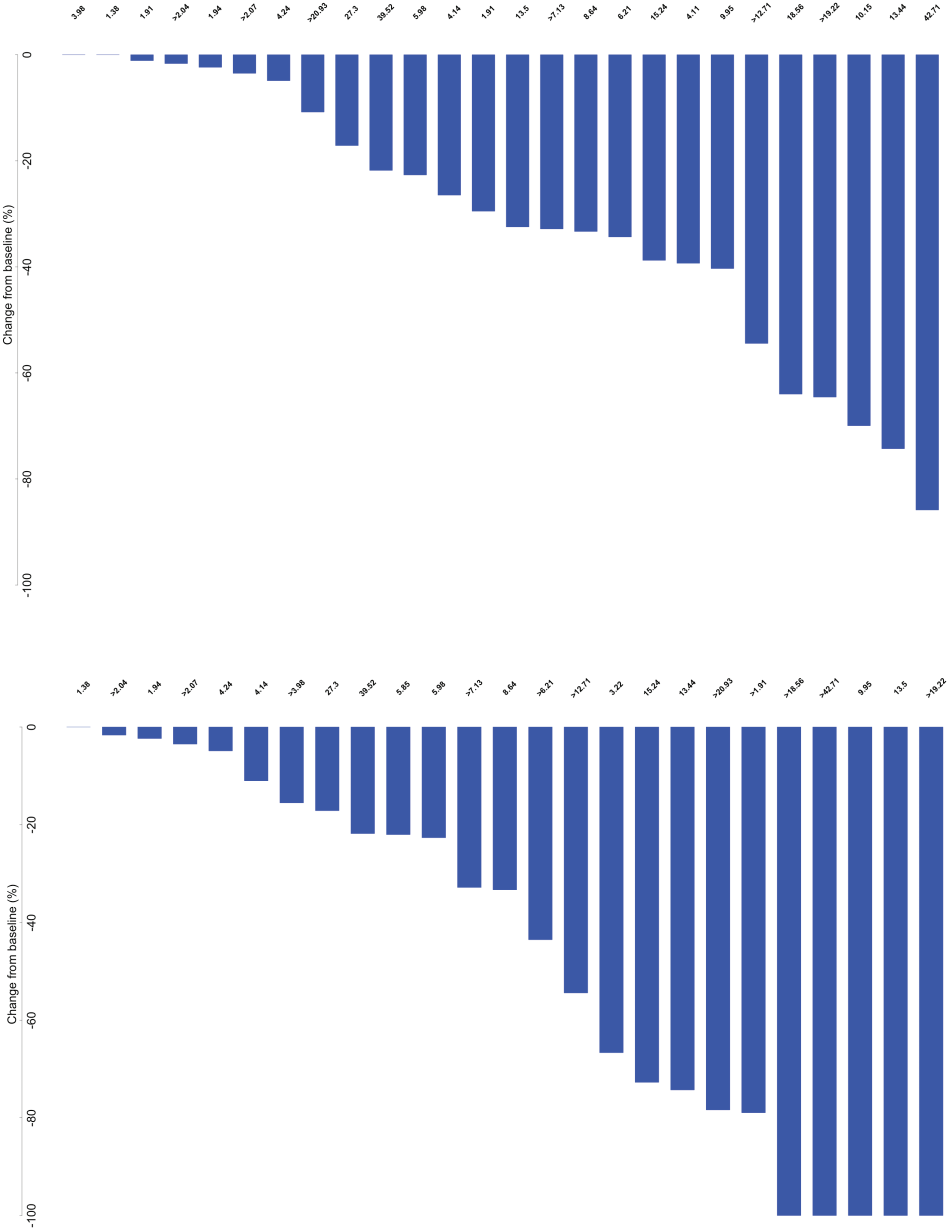
Waterfall plots for target lesion size change from baseline based on RECIST (top) and mRECIST (bottom). PFS and mPFS times are listed for all patients. Patients with right censored PFS or mPFS data are indicated with a ">" in front of the time.

**Figure 4.**
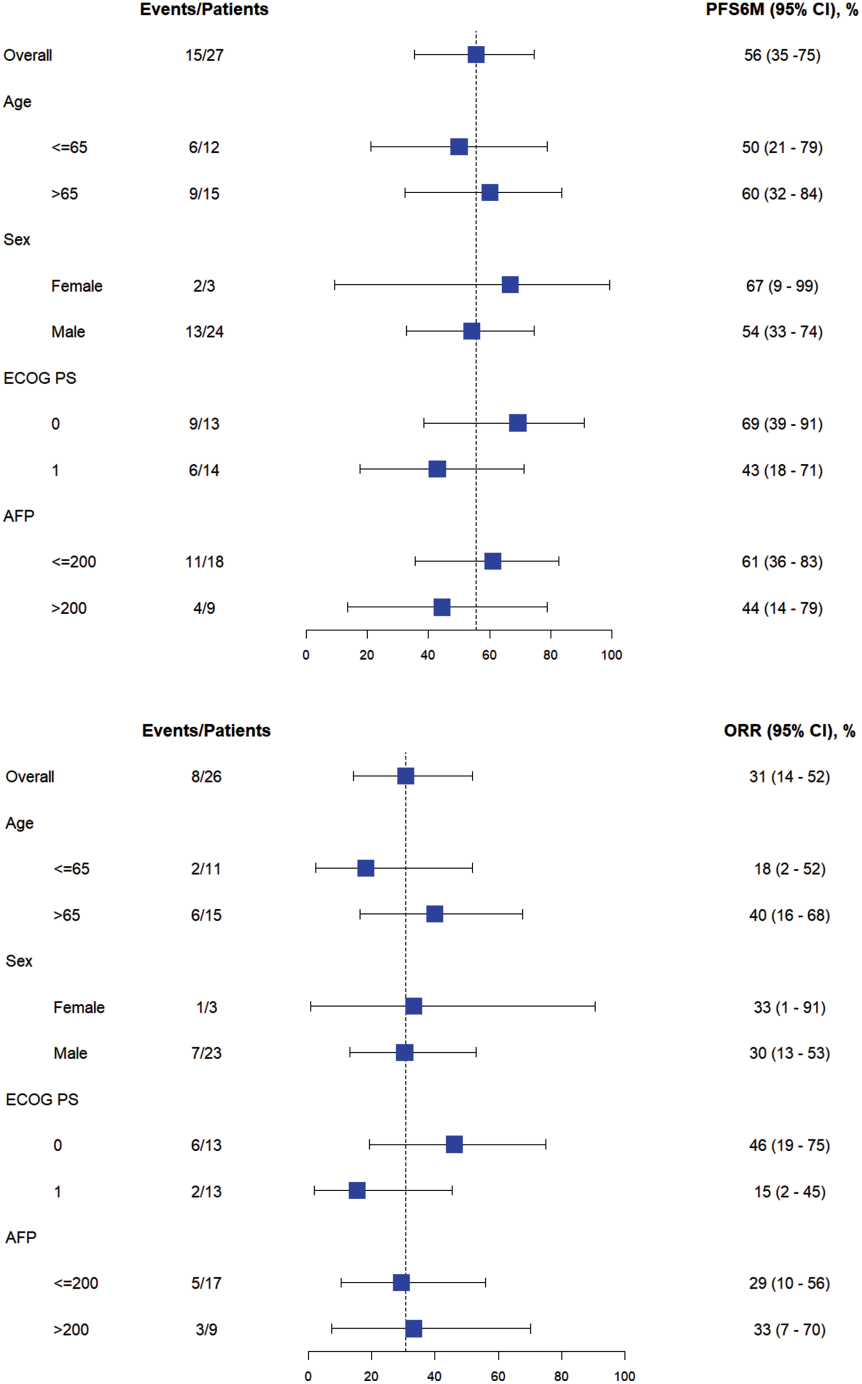
Subgroup analyses of percent of patients progression free at 6 months (top) and objective response as a percent (bottom).

Of the 27 evaluable patients, 15 (55.6%; 95% CI, 35.3-74.5%) were free of progression at 6 months, meeting the study’s primary endpoint. Median PFS was 9.95 months (95% CI, 4.14-15.24); median OS was 27.30 months (95% CI, 10.15-39.52). Among the 26 patients for whom progression was assessed, median TTP was 9.95 months (95% CI, 4.14-15.24; Fig. 2). Per RECIST v1.1 by imaging review, an objective response was observed in 8 of the 26 patients (30.8%; 95% CI, 14.3-51.8%), all with partial response. An additional 14 patients had stable disease for DCR of 84.6% (95% CI, 65.1-95.6%). Progressive disease was best response for 4 of the 26 patients (15.4%; 95% CI, 4.4-34.9%; Tables 3 and 4, Fig. 3). RECIST response data are included in Table 3 and subgroup analyses are similar across patient groups (Fig. 4).

**Table 3.** Responses to pembrolizumab and Y90 combination treatment by RECIST 1.1.

RECIST 1.1 category	Current RECIST response		Best RECIST response	
	*N*	Percent (95% CI)	Counts	Percent (95% CI)
ORR	5	19.2 (6.6%, 39.4%)	8	30.8 (14.3%, 51.8%)
CR	0		0	
PR	5	19.2 (6.6%, 39.4%)	8	30.8 (14.3%, 51.8%)
SD	5	19.2 (6.6%, 39.4%)	14	53.8 (33.4%, 73.4%)
PD	16	61.5 (40.6%, 79.8%)	4	15.4 (4.4%, 34.9%)

**Table 4.** Responses to pembrolizumab and Y90 combination treatment by mRECIST 1.1.

mRECIST	Current mRECIST response		Best mRECIST response	
	*N*	Percent (95% CI)	Counts	Percent (95% CI)
ORR	9	37.5 (18.8%, 59.4%)	9	36.0 (18.0%, 57.5%)
CR	4	16.7 (4.7%, 37.4%)	4	16.0 (4.5%, 36.1%)
PR	5	20.8 (7.1%, 42.2%)	5	20.0 (6.8%, 40.7%)
SD	6	25.0 (9.8%, 46.7%)	14	56.0 (34.9%, 75.6%)
PD	9	37.5 (18.8%, 59.4%)	2	8.0 (1.0%, 26.0%)

mRECIST based on 24 patients: patients 1024 and 1026 are excluded, patients 1006 and 1031 have missing mRECIST, and patient 0007 has NE for mRECIST. Best mRECIST based on 25 patients: patient 0007 has PR for best mRECIST.

### Safety

In all treated patients, median treatment duration was 5.5 months (range, 20 days to 38 months). Treatment-emergent adverse effects were observed in 26 of 27 patients (96.3%); of these, 13 patients (48.1%) had adverse events grade 3 or higher by CTCAE v5.0 including lower lymphocyte counts (19%), elevated bilirubin (11%), and elevated liver function tests (7%). Common events were mostly of grade 1 or 2 in severity. Grade 5 hepatic failure was observed in one patient and deemed related to Y90. Details regarding treatment-emergent adverse effects are presented in Table 5.

**Table 5.** Treatment-emergent adverse events.

Adverse events	Grade 1	Grade 2	Grade 3	Grade 4	Grade 5	Total
Any adverse event	3	10	9	3	1	26
Fatigue	8	8	0	0	0	16
Aspartate aminotransferase increased	7	5	1	0	0	13
Blood bilirubin increased	4	5	2	1	0	12
Abdominal pain	8	3	0	0	0	11
Alanine aminotransferase increased	10	0	1	0	0	11
Lymphocyte count decreased	1	5	4	1	0	11
Nausea	7	2	0	0	0	9
White blood cell decreased	5	4	0	0	0	9
Alkaline phosphatase increased	6	2	0	0	0	8
Anorexia	4	4	0	0	0	8
Anemia	6	0	0	0	0	6
Hypertension	0	3	3	0	0	6
Platelet count decreased	4	0	1	0	0	5
Pruritus	5	0	0	0	0	5
Vomiting	5	0	0	0	0	5
Ascites	1	1	2	0	0	4
Hypoalbuminemia	2	2	0	0	0	4
Neutrophil count decreased	3	1	0	0	0	4
Bloating	3	0	0	0	0	3
Chills	3	0	0	0	0	3
Dry skin	3	0	0	0	0	3
Fever	3	0	0	0	0	3
Hyperthyroidism	3	0	0	0	0	3
Hypomagnesemia	3	0	0	0	0	3
Hypothyroidism	1	2	0	0	0	3
Rash maculo-papular	1	2	0	0	0	3
Arthritis	1	1	0	0	0	2
Blood and lymphatic system disorders—other, specify	1	1	0	0	0	2
Creatinine increased	1	1	0	0	0	2
Dyspnea	2	0	0	0	0	2
Gastrointestinal disorders—other, specify	2	0	0	0	0	2
Hypocalcemia	1	1	0	0	0	2
Hypophosphatemia	0	0	2	0	0	2
Pneumonitis	0	1	1	0	0	2
Portal vein thrombosis	0	2	0	0	0	2
Thrombotic thrombocytopenic purpura	0	2	0	0	0	2
Cardiac disorders—other, specify	1	0	0	0	0	1
Colitis	0	1	0	0	0	1
Constipation	1	0	0	0	0	1
Cough	1	0	0	0	0	1
Diarrhea	1	0	0	0	0	1
Dizziness	1	0	0	0	0	1
Dry eye	1	0	0	0	0	1
Dyspepsia	1	0	0	0	0	1
Edema limbs	1	0	0	0	0	1
Encephalopathy	0	1	0	0	0	1
Endocrine disorders—other, specify	0	1	0	0	0	1
Erythema multiforme	1	0	0	0	0	1
Facial nerve disorder	0	1	0	0	0	1
Facial pain	1	0	0	0	0	1
GGT increased	1	0	0	0	0	1
Hepatic failure	0	0	0	0	1	1
Hepatobiliary disorders—other, specify	1	0	0	0	0	1
Hyperglycemia	0	0	0	1	0	1
Hyperkalemia	1	0	0	0	0	1
Hypernatremia	1	0	0	0	0	1
Hyperuricemia	0	0	1	0	0	1
Hypoglycemia	1	0	0	0	0	1
Hypokalemia	1	0	0	0	0	1
Hyponatremia	1	0	0	0	0	1
Insomnia	1	0	0	0	0	1
Musculoskeletal and connective tissue disorder—other, specify	1	0	0	0	0	1
Myalgia	1	0	0	0	0	1
Neck edema	1	0	0	0	0	1
Pain in extremity	1	0	0	0	0	1
Rash acneiform	0	1	0	0	0	1
Skin and subcutaneous tissue disorders—other, specify	1	0	0	0	0	1
Thromboembolic event	0	1	0	0	0	1
Urticaria	0	1	0	0	0	1
Weight loss	0	1	0	0	0	1

## Assessment, Analysis, and Discussion

**Table:** 

Completion	Study completed
Investigator’s assessment	Active and should be pursued further

**Figure 5.**
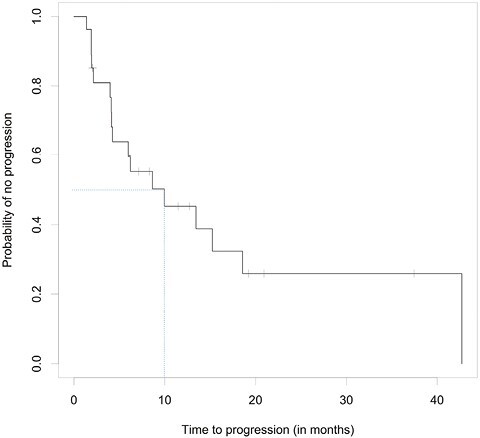
ITT TTP curve: based on 27 patients (Pt 1026 and 1031 had missing progression data). Median TTP is 9.95 months with 95% CI (4.14, 18.56).

**Figure 6.**
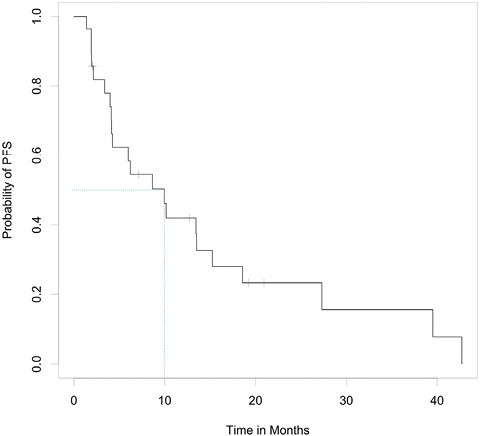
ITT PFS curve: based on 28 patients (Pt 1026 had missing PFS data). Median PFS is 9.95 months with 95% CI (4.11, 15.24).

**Figure 7.**
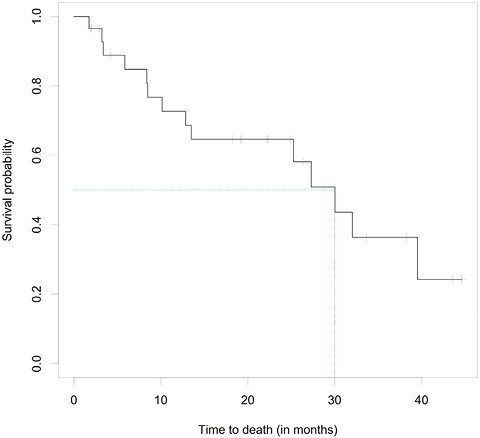
ITT OS curve: based on 29 patients in the ITT population. Median OS is 30 months with 95% CI (12.9, ---). The upper bound was not calculable due to insufficient follow-up for survival.

**Figure 8.**
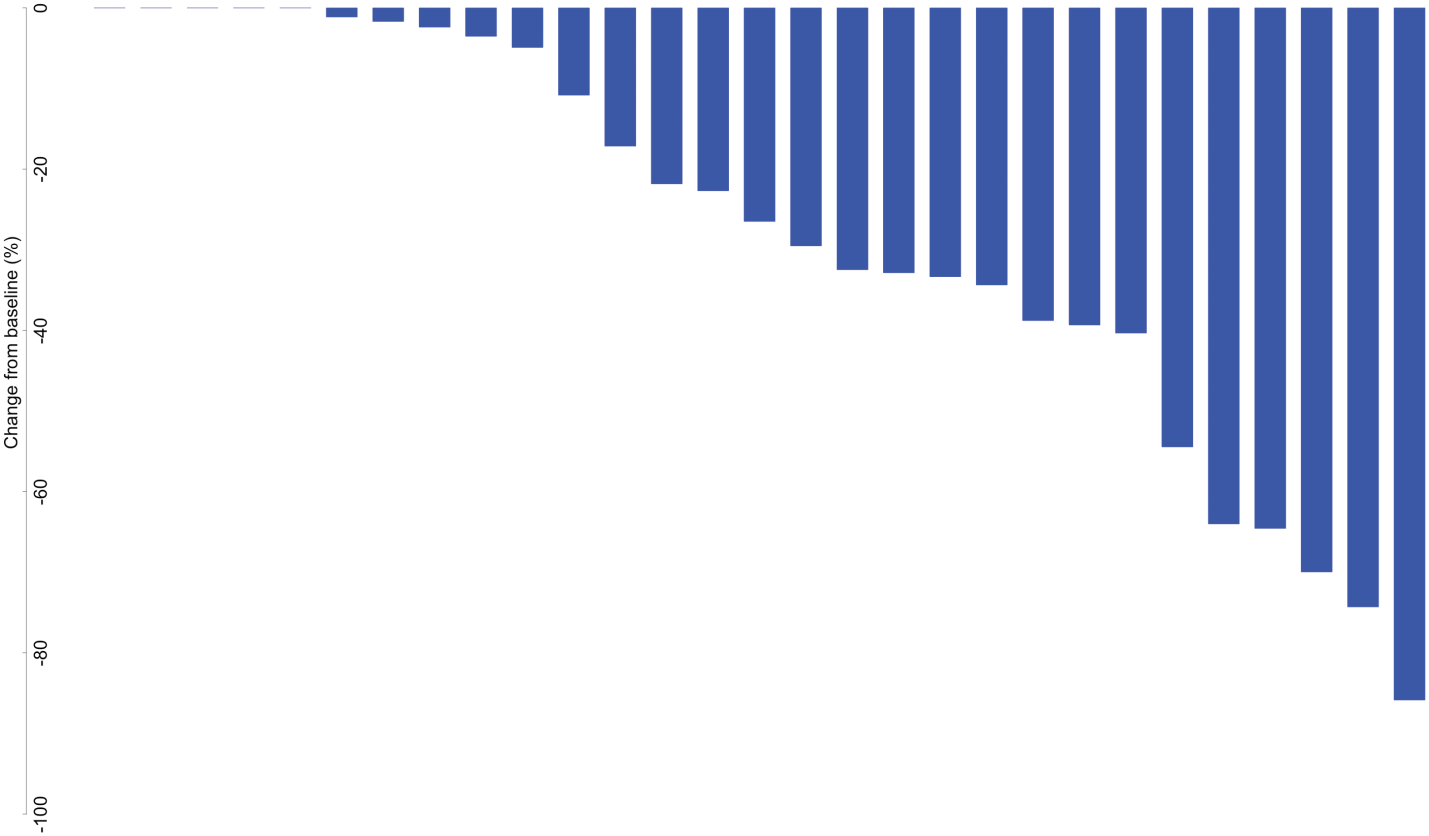
Waterfall plot based on RECIST in the ITT population.

**Figure 9.**
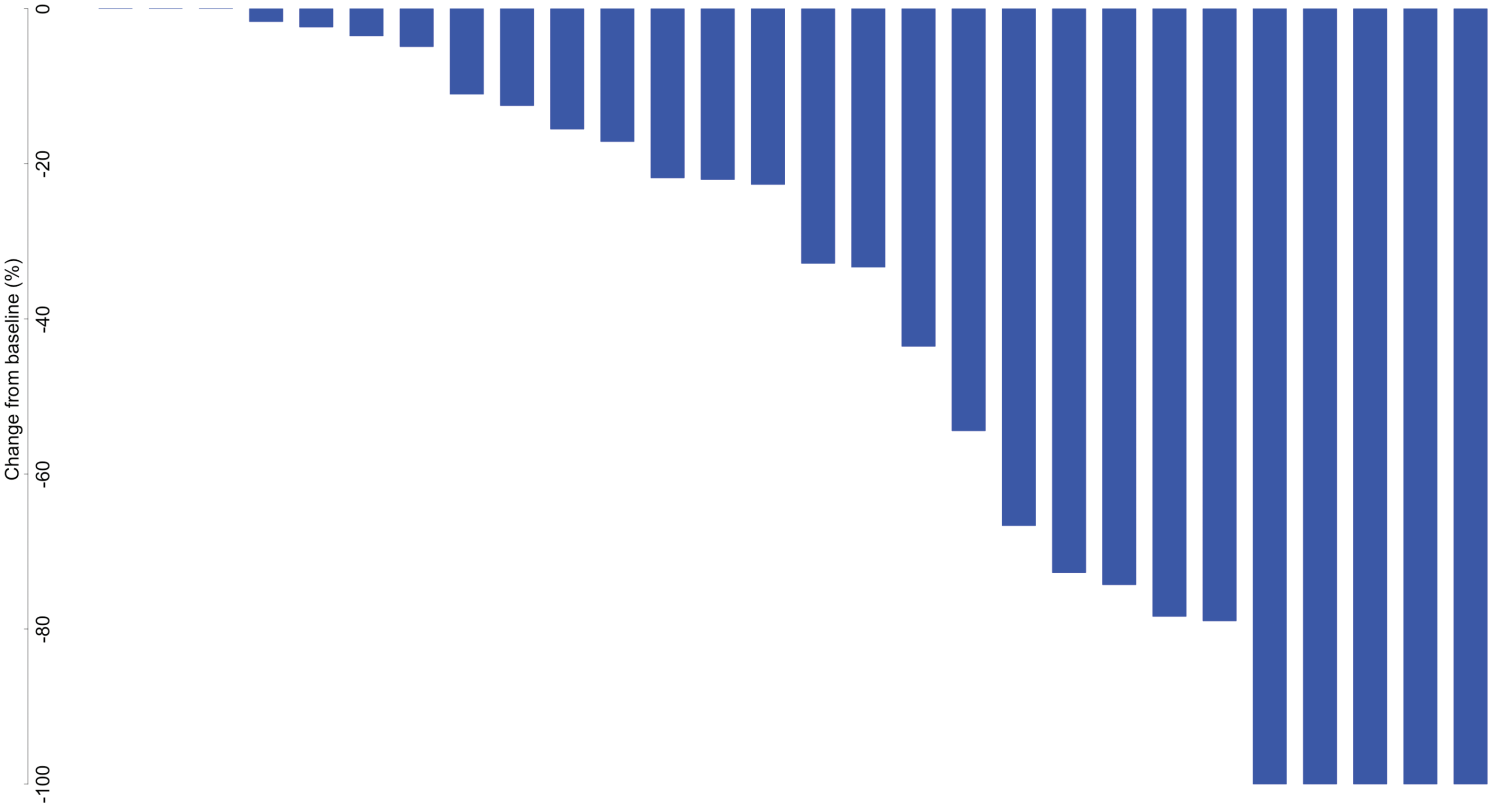
Waterfall plot based on mRECIST in the ITT population.

These reports further support our findings that the addition of pembrolizumab to Y90 radioembolization is a promising and reasonable therapeutic option for patients with poor prognosis HCC. Prior studies evaluating Y90 radioembolization alone in patients with poor prognosis HCC demonstrated a median OS of 13.3 or 6.9 months,^6,7^ while our findings demonstrate that the median OS with the addition of pembrolizumab to be 27.3 months in our treated population. Other historical controls have demonstrated median OS 13.9-14.6 months for pembrolizumab monotherapy^12,17^ and 18.1 months for atezolizumab plus bevacizumab.^18^ While these studies cannot be compared, the large difference in median OS warrants further evaluation in a larger, randomized, 2-arm study. These findings are more striking when evaluating the intent to treat (ITT) population which demonstrated an OS of 30 months and similar rates of response, TTP, and PFS (Tables 6 and 7, Figs. 5-9). These atypical findings can be explained by both patients (1024 and 1026) included in ITT but untreated by our regimen to be “long survivors” with 1024 censored at 24.9 months and 1026 at 22.3 month. Treatment regimens past progression were not collected in this study but given the established benefit checkpoint blockade in later settings, further evaluation in a larger study is warranted to determine if earlier treatment with checkpoint blockade similar to our study is beneficial to patient survival.

**Table 6.** ITT RECIST 1.1.

RECIST 1.1 category	Current RECIST response		Best RECIST response	
	*N*	Percent (95% CI)	*N*	Percent (95% CI)
ORR	5	18.5 (6.3%, 38.1%)	8	29.6 (13.8%, 50.2%)
CR	0		0	
PR	5	18.5 (6.3%, 38.1%)	8	29.6 (13.8%, 50.2%)
SD	5	18.5 (6.3%, 38.1%)	14	51.9 (31.9%, 71.3%)
PD	17	63 (42.4%, 80.6%)	5	18.5 (6.3%, 38.1%)

Response is based on 27 patients because patients 1024 and 1026 were missing response data.

**Table 7.** ITT mRECIST.

mRECIST	Current mRECIST response		Best mRECIST response	
	*N*	Percent (95% CI)	Counts	Percent (95% CI)
ORR	9	36 (18%, 57.5%)	9	34.6 (17.2%, 55.7%)
CR	4	16 (4.5%, 36.1%)	4	15.4 (4.4%, 34.9%)
PR	5	20.0 (6.8%, 40.7%)	5	19.2 (6.6%, 39.4%)
SD	7	28.0 (12.1%, 49.4%)	15	57.7 (36.9%, 76.6%)
PD	9	36 (18%, 57.5%)	2	7.7 (0.9%, 25.1%)

Response based on 25 patients. Patients 1006, 1026, 1027, and 1031 have missing mRECIST. ITT Best mRECIST Based on 26 patients. Patients 1006, 1026, and 1031 have missing mRECIST.

Previous studies have shown that Y90 radioembolization has shown benefit in patients with early intermediate stage HCC,^19^ but not in patients with localized, poor prognosis HCC.^6,7^ More recently, other studies have shown that immune checkpoint inhibition as monotherapy or as combination therapy can provide a clinical benefit for patients with advanced HCC. CheckMate-040^8,10^ and CheckMate-459^9^ demonstrated benefit of nivolumab or nivolumab plus ipilimumab in the treatment of advanced HCC. KEYNOTE-224 (11,13), KEYNOTE-240 (12), and KEYNOTE-394 (14) demonstrated efficacy of pembrolizumab monotherapy in advanced HCC. IMbrave150 demonstrated benefit of atezolizumab plus bevacizumab in locally advanced metastatic or unresectable HCC^15^; ORIENT-32 examined sintilimab plus a bevacizumab biosimilar (IBI305) as first-line treatment for unresectable HBV-associated hepatocellular carcinoma.^16^ Systemic therapies in combination with Y90 radioembolization have been explored in patients with advanced HCC as well. In another recent single-center prospective study also involving patients with advanced HCC and Child-Pugh A cirrhosis, 36 patients were given Y90 radioembolization followed by nivolumab with 30.6% objective response rate,^20^ similar to the 30.8% observed in our patients. Other historical controls demonstrate ORR 12.7%-18.3% for pembrolizumab monotherapy,^17^ ORR 20% for nivolumab monotherapy,^8^ and ORR 30% for atezolizumab plus bevacizumab.^18^ However, a multicenter study involving pembrolizumab in combination with Y90 radioembolization has not previously been performed.

The synergistic effect between radiotherapy and immunotherapy has been explored in recent preclinical and clinical models. The ability of stereotactic radiotherapy to induce endogenous antigen-specific immune responses significantly increased when combined with anti-PD-1 therapy in murine models of melanoma and breast carcinoma.^21^ In murine models of HCC, radiation upregulated PD-L1 expression in tumor models via IFN-γ/STAT3 signaling, resulting in significantly suppressed tumor growth and improved survival in mice receiving combination anti-PD-L1 and radiation therapy.^22^ Systemic immune activation was described in analysis of the immune landscape of tumor-infiltrating lymphocytes from patients before and after receiving Y90 radioembolization.^23^ This was further elucidated in patients with intermediate-advanced HCC receiving Y90 radioembolization, in which tumor irradiation induced an altered adaptive and innate response that peaked 1 month after treatment and diminished significantly at 3 and 6 months. Moreover, many of the induced CD4+ and CD8+ T cells expressed high levels of inhibitory checkpoint markers PD-1 and LAG3, from which the investigators speculate that immune checkpoints administered after Y90 radioembolization would enhance both the immunological and clinical efficacy of irradiation.^24^

Our results suggest that pembrolizumab administration in combination with glass Y90 radioembolization was safe and tolerable. This combination also provided durable antitumor activity, promising PFS and OS in patients with advanced, poor prognosis HCC who had not previously received anti-PD-1, anti-PD-L1, or anti-CTLA-4 antibody therapy. These data demonstrating tolerability and improved OS suggest that patients with poor prognosis HCC may have similar risk stratification as metastatic patients warrant further evaluation.

## Supplementary Material

oyad331_suppl_Supplementary_Appendix

## Data Availability

The data underlying this article were provided by Merck under licence/by permission. Data will be shared on request to the corresponding author with permission of Merck.
