# Supplementary material for: A Pilot Study of Pembrolizumab in Combination With Y90 Radioembolization in Subjects With Poor Prognosis Hepatocellular Carcinoma
Source: Oncologist. 2024 Feb 7;29(3):270–e413. doi: 10.1093/oncolo/oyad331 (PMC10911903; doi:10.1093/oncolo/oyad331)
Supplement: oyad331_suppl_Supplementary_Appendix [file oyad331_suppl_supplementary_appendix.zip › appendix_inclusion_exclusion.docx]

**APPENDIX – ELIGIBILITY CRITERIA**

Inclusion Criteria

Subject must meet all of the following applicable inclusion criteria to participate in this study:

1. Written informed consent and HIPAA authorization for release of personal health information prior to registration. **NOTE:** HIPAA authorization may be included in the informed consent or obtained separately.
2. Age ≥ 18 years at the time of consent.
3. ECOG Performance Status of 0-1.
4. Locally advanced HCC as defined by: 1) tissue diagnosis **OR** 2) alpha-fetoprotein (AFP) > 400 ng/mL with compatible mass on contrast-enhanced imaging **OR** 3) compatible mass on dual phase CT or dynamic contrast enhanced MRI demonstrating both arterial hypervascularity and delayed washout.
5. Hepatopulmonary shunting < 20% as documented via hepatic artery perfusion study.
6. No evidence of extrahepatic metastatic disease.
7. Subjects must be considered poor prognosis by the following parameters: 1) right or left portal vein involvement (**NOTE:** subjects with main portal vein involvement are excluded), 2) multi-focal disease (more than 3 tumors regardless of size) **AND/OR** 3) diffuse disease considered amenable to liver directed therapy.
8. Subjects with chronic infection by HCV who are untreated or who failed previous therapies for HCV are allowed on study. In addition, subjects with successful HCV treatment (defined as sustained virologic response [SVR] 12 or SVR 24) are allowed as long as patients are not actively receiving anti-HCV treatment at the time of study enrollment. Investigators can stop anti-HCV treatment at their discretion prior to enrolling patients on study.
9. If active HBV, viral load must be <100IU/mL; if active HBV, subjects must be on anti-viral medication for ≥ 3 months prior to study registration and remain on the same anti-viral regimen throughout study treatment. **NOTE:** those subjects who are positive for Hepatitis B core antibody (anti-HBc), negative for Hepatitis B surface antigen (HBsAg) and negative for Hepatitis B surface antibody (anti-HBs), and have an HBV viral load <100 IU/mL do not require HBV anti-viral prophylaxis.
10. Not eligible for surgical resection or liver transplant or have refused such procedures.
11. All disease must be amenable to embolization in one or two procedures.
12. Childs-Pugh Cirrhotic Status A or B with a maximum score of 7.
13. No evidence of clinically apparent ascites or active encephalopathy, and/or varices that have not been treated. Subjects with controlled ascites or encephalopathy are eligible so long as they meet Childs-Pugh score criterion. Please note that controlled ascites and encephalopathy require scores of 2 each when calculating the C-P score.
14. No prior Y90 radioembolization for HCC is permitted. Therapies below are allowed but must be completed 4 weeks prior to baseline scan.

- Prior TAE or TACE
- One treatment of stereotactic body radiation therapy (SBRT)
- Liver resection
- Ablation therapy

1. Demonstrate adequate organ function as defined in the table below. All screening labs to be obtained within 28 days prior to registration.

| **System** | **Laboratory Value** |
| --- | --- |
| **Hematological** ^1^Without erythrocyte stimulating agent or transfusion within 7 days of screening | |
| Absolute Neutrophil Count (ANC) | ≥ 1.5 × 10^9^/L |
| Hemoglobin (Hgb)^1^ | ≥ 9 g/dL |
| Platelet Count | ≥ 60 × 10^9^/L |
| **Renal** ^2^Cockcroft-Gault formula will be used to calculate creatinine clearance | |
| Creatinine **OR** | < 1.5 × ULN |
| Calculated creatinine clearance^2^ | ≥ 60 cc/min |
| **Hepatic** | |
| Bilirubin | < 2.0 × ULN |
| Aspartate aminotransferase (AST) | ≤ 5 × ULN |
| Alanine aminotransferase (ALT) | ≤ 5 × ULN |
| **Coagulation** | |
| International Normalized Ratio (INR) or Prothrombin Time (PT) or Activated Partial Thromboplastin Time (aPTT) | ≤ 1.5 |

1. Females of childbearing potential must have a negative serum pregnancy test within 72 hours prior to registration. **NOTE:** Females are considered of child bearing potential unless they are surgically sterile (have undergone a hysterectomy, bilateral tubal ligation, or bilateral oophorectomy at least 6 weeks prior to study registration) or they are naturally postmenopausal for at least 12 consecutive months without an alternative medical cause.
2. Females of childbearing potential and males must be willing to abstain from heterosexual activity* or to use effective methods of contraception from the time of informed consent until 120 days after treatment discontinuation. Acceptable contraception methods can be comprised of an intrauterine device (IUD), vasectomy of a female subject’s male partner, contraceptive rod implanted into the skin, **or** use of **two** of the following: diaphragm with spermicide (cannot be used in conjunction with cervical cap/spermicide), cervical cap with spermicide (nulliparous women only), contraceptive sponge (nulliparous women only), male condom or female condom (cannot be used together), hormonal contraceptive. *Abstinence is acceptable if this is the usual lifestyle and preferred contraception for the subject.
3. As determined by the enrolling physician or protocol designee, ability of the subject to understand and comply with study procedures for the entire length of the study.
4. Is willing to undergo a mandatory pre-treatment research biopsy at the centers participating in research biopsies. A cohort of 10 patients from the University of North Carolina (UNC) will undergo a post-treatment research biopsy after 3 cycles of pembrolizumab.

Exclusion Criteria

Subjects meeting any of the criteria below may not participate in the study:

1. Is currently participating and receiving study therapy or has participated in a study of an investigational agent and received study therapy or used an investigational device within 4 weeks of study registration.
2. Diagnosis of immunodeficiency or is receiving systemic steroid therapy (other than oral contraceptives) or any other form of immunosuppressive therapy within 7 days prior to registration.
3. Active autoimmune disease that has required systemic treatment in the past 2 years (i.e. with use of disease modifying agents, corticosteroids or immunosuppressive drugs). Replacement therapy (e.g. thyroxine, insulin, or physiologic corticosteroid replacement therapy for adrenal or pituitary insufficiency) is not considered a form of systemic treatment.
4. Known history of active TB.
5. Hypersensitivity to pembrolizumab or any of its excipients.
6. Has had a prior anti-cancer monoclonal antibody (mAb) within 4 weeks prior to registration or who has not recovered (i.e., ≤ Grade 1 or baseline) from adverse events due to agents administered > 4 weeks prior.
7. Has had prior chemotherapy, targeted small molecule therapy or radiation therapy within 2 weeks prior to registration, or who has not recovered (i.e., ≤ Grade 1 or baseline) from AEs due to previously administered agents.
8. If had major surgery, subject must have recovered adequately from the toxicity and/or complications from the intervention prior to study registration.
9. Complete portal vein occlusion.
10. Vascular abnormalities or bleeding diathesis that indicates hepatic artery catheterization is contraindicated.
11. Received prior therapy with an anti-PD-1, anti-PD-L1, or anti-CTLA-4 antibody.
12. Known history of HIV.
13. Untreated active HBV.
14. Dual infection with HBV/HCV or other hepatitis combinations at study entry.
15. Known history of, or any evidence of active, non-infectious pneumonitis.
16. History of organ or stem cell transplantation including previous history of liver transplantation.
17. Active infection requiring systemic therapy.
18. Pregnant or breastfeeding (**NOTE:** breast milk cannot be stored for future use while the mother is being treated on study).
19. Has history or current evidence of any condition, therapy or laboratory abnormality that may confound results or interfere with subject’s participation in the trial.
20. Known additional malignancy that is active and/or progressive requiring treatment; exceptions include basal cell or squamous cell skin cancer, in situ cervical or bladder cancer, or other cancer for which the subject has been disease-free for at least three years.
21. Has received a live vaccine within 30 days of planned start of study therapy.
